# Patterns of weight change associated with disease diagnosis in a national sample

**DOI:** 10.1371/journal.pone.0207795

**Published:** 2018-11-26

**Authors:** Yana C. Vierboom, Samuel H. Preston, Andrew Stokes

**Affiliations:** 1 Department of Sociology and Population Studies Center, University of Pennsylvania, Philadelphia, PA, United States of America; 2 Department of Global Health, Boston University School of Public Health, Boston, MA, United States of America; Indiana University Purdue University at Indianapolis, UNITED STATES

## Abstract

**Background:**

The incidence and/or diagnosis of a major disease may activate weight change. Patterns of weight change associated with diagnoses have not been systematically documented.

**Methods:**

We use data on adults ages 30+ in the National Health and Nutrition Examination Survey (NHANES) from 1999–2014. Self-reported current weight and weight one year prior are used to estimate percent weight change in the last year. We use self-reported data on arthritis, diabetes, cancer, cardiovascular disease, liver conditions, and respiratory disease diagnoses to compare weight change among individuals never diagnosed with these conditions, individuals diagnosed 0–1 years ago, and individuals diagnosed 2+ years ago. Multinomial logistic regressions adjust for the presence of multiple conditions.

**Results:**

17.7% of the adult population experienced weight loss of 5.0% or more in the year prior to survey. Individuals diagnosed with any of the conditions were less likely to maintain their weight than those without a diagnosis. Arthritis, diabetes, cancer, cardiovascular disease, and liver conditions were associated with net weight loss, whereas respiratory diseases were associated with higher probabilities of both losing and gaining weight. Among those losing 10% or more, 56.7% had been diagnosed with one of the conditions. Cancer was associated with the highest probability of unintentional weight loss and diabetes with the highest probability of intentional weight loss.

**Conclusions:**

Disease-associated weight changes leave a distinct imprint on patterns of weight change in the population. Individuals losing at least 10% of their weight in the last year have likely been diagnosed with one of the six conditions.

## Introduction

Many factors determine changes in an individual’s weight from year to year. With varying degrees of success, a person may voluntarily choose to lose or gain weight. Weight change may also be involuntary, a result of changes in appetite, food availability, prescription medications, mobility limitations, or changes in basal metabolic rate.[1,2]

The incidence and/or diagnosis of a major disease may activate any or all of these mechanisms. For example, a diagnosis of diabetes is likely to encourage voluntary efforts to lose weight.[3,4] Poor glycemic control in diabetes may lead to involuntary weight loss.[5] Chemotherapy used to treat cancer is often associated with loss of appetite.[6] Both cancer and chronic obstructive pulmonary disease are associated with the development of cachexia through increased metabolic demands, among other routes.[7,8] Arthritis often limits physical activity and may be associated with weight change.[9] Efforts to reduce smoking after diagnosis of chronic bronchitis and other smoking-related illnesses may indirectly lead to weight gain through changes in appetite and metabolism.[10,11]

Patterns of weight change associated with diagnoses of major diseases have not been systematically documented in a national sample of US adults. In this paper, we use data from the National Health and Nutrition Examination Survey to document weight change in the year preceding the survey as a function of recent, as well as more distant, disease diagnosis. The disease categories of interest are arthritis, diabetes, cancer, cardiovascular disease, liver disease, and respiratory disease. Special attention is paid to the distinction between voluntary and involuntary weight loss. Whereas involuntary weight loss represents a useful prognostic indicator of disease onset and severity, voluntary weight loss may be indicative of behavioral modification in response to the disease diagnosis and encounter with the health care system. Comparisons by length of time since diagnosis help to reveal the extent to which patterns of weight change are maintained or dissipate over time. The patterns that we describe provide a set of provisional standards that can help to inform clinicians about weight changes to be expected when patients are diagnosed with one of the diseases that we investigate. We demonstrate the major role that disease incidence is playing in US patterns of weight change, a role that has not previously been identified.

## Data and methods

We used data from the National Health and Nutrition Examination Survey (NHANES), pooled across eight waves from 1999–2014.[12] NHANES is an annual cross-sectional health survey that is representative of the non-institutionalized US population. Our sample was restricted to non-pregnant individuals ages 30 and above not missing data on self-reported weight at survey and one year prior, disease diagnosis, age at diagnosis, and model covariates. Roughly 95% of respondents in our sample were medically examined as part of the survey, which includes being weighed. To minimize possible bias associated with weight misreporting, we excluded 1,673 examined people who had a discrepancy between self-reported and measured weight of 10% or more. The reliability of weight self-reporting could not be assessed for the remaining 5% (1,584) of otherwise-eligible respondents who completed the questionnaire portion of the survey but were not medically examined and were thus missing information on measured weight. We retained these individuals. The final sample size was 31,860.

All waves asked respondents their current weight as well as their weight one year ago, which we used to construct a continuous variable for percent weight change in the last year. Individuals who were pregnant one year ago were asked to report their weight prior to pregnancy. For descriptive purposes, we divided weight change into seven categories: three for weight loss (2.5–4.9%, 5–9.9%, and 10% or greater), one for no change (between loss of 2.5% and gain of 2.5%), and three for weight gain (2.5–4.9%, 5–9.9%, and 10% or greater). We used the continuous measure to estimate the mean percent change.

Respondents were also asked whether “a doctor or other health professional ever told you that you had” certain medical conditions or diseases. We used these data to create six disease-specific categories: arthritis, diabetes, liver condition, cancer, cardiovascular disease (CVD), and respiratory disease. The last three categories are combinations of several diagnosis questions asked in the survey. Cancer includes all cancers except non-melanoma skin cancer, a common condition which is not likely to be linked to weight change. CVD includes congestive heart failure, coronary heart disease, angina or angina pectoris, and stroke. Respiratory disease includes emphysema and chronic bronchitis. In a preliminary analysis, we categorized lung cancer as a respiratory disease, rather than as a cancer, with little effect on the results. Although there are other conditions that may be associated with weight change, NHANES does not consistently include information on their diagnosis; this analysis was therefore limited to diagnosis of the six categories listed above.

We treated diagnoses independently of one another; a respondent who reported multiple conditions was counted in multiple categories. We created a seventh category, “none”, for respondents who reported never having been diagnosed with any of the six conditions.

Respondents who indicated a diagnosis were additionally asked the age at which they first received the diagnosis. For those reporting several conditions within the same category, such as multiple cancers, we used the earliest diagnosis. We subtracted respondent’s age at diagnosis from respondent’s current age to create a categorical variable for years since diagnosis (0–1 years, and 2+ years).

Respondents who reported losing at least 10 pounds in the last year were asked whether their weight loss was intentional. We used these data to identify the frequency of intentional and unintentional weight loss of at least 10% for people in different diagnostic categories. Only three individuals who lost at least 10% of their body weight did not lose at least 10 pounds, so were not asked about intention. They are included as not losing 10% in the analysis of intention.

We estimated multinomial logistic regressions predicting the probability of gaining or losing 10% of body weight in the year preceding the survey. We used the six disease categories as predictor variables. Within each category, we differentiated between those diagnosed 0–1 years ago and those diagnosed 2+ years ago; those who have never been diagnosed with the given disease serve as the reference category. These regressions allow us to control the presence of other disease categories when studying the relation between a particular diagnosis and weight change. All regressions adjusted for age, sex, and the average of weight at survey and one year prior. At a second stage, we also adjusted for other variables that may be related to weight change: smoking status (never, former, current), race/ethnicity (Hispanic, non-Hispanic white, non-Hispanic black, and other), and educational attainment (non-graduate of high school, high school graduate, some college, and BA+).

We also investigated whether there was a significant interaction between recent diagnosis and age in their effect on weight loss or weight gain. To do so, we created six multiplicative variables combining each of the six recent diagnoses with age. Similarly, we investigated whether there was a significant interaction between recent diagnosis and mean weight as predictors of weight loss or weight gain. In a sensitivity analysis examining possible interactions between diagnoses, we included dummy variables indicating whether a respondent had ever been diagnosed with some of the most common comorbidities (arthritis and cardiovascular disease; cardiovascular disease and diabetes; arthritis and diabetes), separately for each comorbidity.

We accounted for the complex sampling design of NHANES using the *svy* command in Stata version 15 (StataCorp). All analyses were weighted using weights for the interviewed sample.

## Results

Table 1 presents the distribution of weight change in the past year for the entire sample, for people diagnosed 0–1 years ago with one of the diseases, and for people who have never received one of the six diagnoses. For those never diagnosed with one of the diseases, the mean percentage change in weight over the last year was positive at 0.55%. However, when those diagnosed are included in the sample as a whole, the mean weight change declined to 0.16%. 17.7% of all adults lost at least 5% of their body weight in the year prior to survey and 7.5% of individuals lost 10% or more of their body weight.

**Table 1:** Percent weight change in the last year for those diagnosed 0–1 year ago. Adults 30+, NHANES 1999–2014. 95% confidence intervals in brackets.

		Diagnosis						
	
	Entire sample	None^a^	Arthritis	Cancer^b^	CVD^b^	Diabetes	Liver condition	Respiratory disease^b^
								
**Mean % change**	0.16	0.55	-0.48	-2.66	-1.01	-2.68	-1.74	2.33
								
**Weight loss (%)**								
**-10+ %**	7.49 [7.06,7.92]	5.73 [5.23,6.23]	9.89 [7.92,11.85]	18.37 [11.96,24.79]	15.05 [11.67,18.44]	18.31 [13.64,22.97]	18.94 [12.22,25.66]	13.47 [7.54,19.41]
**-5 to -9.9%**	10.21 [9.84,10.59]	9.17 [8.62,9.71]	11.91 [9.58,14.24]	13.56 [8.78,18.33]	12.71 [9.62,15.80]	19.77 [15.24,24.30]	12.73 [7.69,17.77]	7.98 [4.17,11.79]
**-2.5 to -4.9%**	7.37 [6.98,7.76]	7.34 [6.82,7.86]	7.06 [5.44,8.68]	6.76 [4.18,9.34]	8.93 [6.14,11.73]	11.25 [7.51,15.00]	8.33 [4.72,11.95]	5.82 [1.60,10.03]
								
**No change (%)**								
**-2.4 to 2.4%**	49.16 [48.29,50.03]	51.07 [50.07,52.07]	46.84 [43.51,50.17]	38.70 [32.47,44.94]	40.02 [36.08,43.95]	29.29 [24.26,34.31]	34.89 [26.98,42.80]	37.86 [29.85,45.86]
								
**Weight gain (%)**								
**2.5 to 4.9%**	8.73 [8.33,9.13]	9.37 [8.81,9.92]	9.49 [7.31,11.66]	6.83 [3.37,10.29]	5.08 [3.00,7.16]	4.95 [2.46,7.44]	6.09 [1.75,10.43]	9.07 [4.50,13.63]
**5 to 9.9%**	9.57 [9.08,10.07]	10.03 [9.44,10.63]	7.97 [6.37,9.57]	10.15 [5.65,14.65]	9.52 [6.72,12.32]	9.40 [5.74,13.07]	10.48 [4.85,16.11]	7.60 [3.51,11.68]
**10+ %**	7.47 [7.04,7.89]	7.30 [6.77,7.83]	6.85 [4.96,8.73]	5.63 [2.17,9.08]	8.69 [5.91,11.46]	7.03 [3.80,10.26]	8.53 [3.83,13.24]	18.21 [10.15,26.27]
								
**N**	31,860	16,439	1,577	393	693	525	241	258

a. None of the included conditions diagnosed prior to study.

b. Cancer includes all cancers except non-melanoma skin cancers. CVD (cardiovascular disease) includes congestive heart failure, coronary heart disease, angina or angina pectoris, and stroke. Respiratory disease includes emphysema and chronic bronchitis.

Table 1 also shows that those diagnosed in the past 0–1 years with arthritis, cancer, cardiovascular disease, diabetes, and liver conditions lost weight, on average, over the past year. Of those diagnosed with diabetes, 38.1% lost at least 5% of their body weight over the past year. The equivalent percentage for cancer was 31.9%, 31.7% for liver conditions, and 27.8% for cardiovascular disease. These large fractions of weight losers are 1.9–2.6 times greater than the percentage of weight losers among those with no diagnosed condition, 14.9%. The percentage losing at least 10% of their body weight for these four conditions ranged from 15.1% to 18.9%, compared to only 5.7% for those with no diagnosed conditions.

The main exception to the pattern of weight loss associated with disease diagnosis is respiratory disease. Table 1 shows that those recently diagnosed with respiratory disease gained, on average, 2.33% of body weight over the past year, including 18.2% who gained more than 10%. This percentage is much higher than for any other disease and 2.5 times that of people with no diagnosis. One factor that is likely to be involved in the weight gain of people diagnosed with respiratory disease is smoking cessation. If the sample is limited to those who never smoked, the mean percentage weight gain for those with a recent diagnosis of respiratory disease is 0.69%, compared to 2.33% for the whole sample (results not shown).

Whether contributing to weight loss, weight gain, or both, disease diagnosis is disruptive of normal patterns of weight maintenance. 51.1% of the sample with no disease diagnosis maintained a weight within 2.5% of their weight one year ago. In contrast, the percentage of those with a diagnosis who maintained their weight ranged from 29.3% (diabetes) to 46.8% (arthritis).

Table 2 presents data equivalent to Table 1 but for people diagnosed 2+ years before survey. In general, trends observed for more recent diagnoses continue 2+ years after diagnosis, though at a slower pace. Weight loss of 10% or more is 1.7–2.5 times more frequent among those with any diagnosis at duration 2+ than it is among those with no diagnosis. By far the largest incidence of weight loss at 2+ years is observed for those diagnosed with diabetes, among whom 28.0% lost at least 5% of body weight in the past year and 14.3% lost at least 10%. Weight gain remains more common among those with respiratory diagnoses than in other diagnostic categories.

**Table 2:** Percent weight change in the last year for those diagnosed 2+ years ago. Adults 30+, NHANES 1999–2014. 95% confidence interval in brackets.

		Diagnosis						
	
	Entire sample	None^a^	Arthritis	Cancer^b^	CVD^b^	Diabetes	Liver condition	Respiratory disease^b^
								
**Mean % change**	0.16	0.55	-0.40	-0.52	-0.65	-1.41	-0.42	0.31
								
**Weight loss (%)**								
**-10+ %**	7.49 [7.06,7.92]	5.73 [5.23,6.23]	9.84 [8.94,10.73]	9.98 [8.09,11.88]	11.35 [9.88,12.81]	14.29 [12.87,15.71]	10.58 [8.22,12.94]	11.00 [9.43,12.56]
**-5 to -9.9%**	10.21 [9.84,10.59]	9.17 [8.62,9.71]	11.74 [10.95,12.53]	9.52 [7.78,11.27]	10.86 [9.49,12.22]	13.74 [12.18,15.30]	10.48 [8.10,12.86]	11.38 [9.66,13.09]
**-2.5 to -4.9%**	7.37 [6.98,7.76]	7.34 [6.82,7.86]	7.35 [6.62,8.08]	8.40 [6.74,10.07]	8.28 [7.15,9.41]	8.48 [7.36,9.60]	5.36 [3.72,7.00]	5.97 [4.71,7.23]
								
**No change (%)**								
**-2.4 to 2.4%**	49.16 [48.29,50.03]	51.07 [50.07,52.07]	46.81 [45.19,48.42]	47.38 [44.26,50.49]	47.02 [44.68,49.36]	42.21 [40.06,44.35]	47.98 [44.08,51.87]	41.21 [38.30,44.12]
								
**Weight gain (%)**								
**2.5 to 4.9%**	8.73 [8.33,9.13]	9.37 [8.81,9.92]	7.22 [6.57,7.86]	9.04 [7.39,10.69]	7.37 [6.31,8.44]	6.42 [5.36,7.48]	7.25 [5.02,9.48]	8.35 [6.71,10.00]
**5 to 9.9%**	9.57 [9.08,10.07]	10.03 [9.44,10.63]	9.57 [8.68,10.45]	8.57 [6.71,10.43]	7.50 [6.33,8.67]	7.10 [6.13,8.07]	11.01 [8.87,13.16]	11.29 [9.64,12.94]
**10+ %**	7.47 [7.04,7.89]	7.30 [6.77,7.83]	7.49 [6.76,8.21]	7.10 [5.23,8.98]	7.63 [6.39,8.86]	7.76 [6.47,9.05]	7.33 [5.25,9.42]	10.81 [9.08,12.53]
								
**N**	31,860	16,439	8,402	1,929	3,065	3,688	1,044	2,129

a. None of the included conditions diagnosed prior to study.

b. Cancer includes all cancers except non-melanoma skin cancers. CVD (cardiovascular disease) includes congestive heart failure, coronary heart disease, angina or angina pectoris, and stroke. Respiratory disease includes emphysema and chronic bronchitis.

Table 3 shows the extent of weight loss of at least 10%, by intentionality, for people diagnosed with each of the conditions identified. In the absence of disease, extensive unintentional weight loss is very uncommon; among those with no diagnosis, only 1.5% experienced unintentional weight loss of at least 10% in the year before the survey. The prevalence of unintentional weight loss was much greater than 1.5% for *all* diagnoses at *both* durations since diagnosis. Those diagnosed with cancer at durations 0–1 show the highest incidence of unintentional weight loss, at 11.3%. With the exception of arthritis, unintentional weight loss was more prevalent among those diagnosed more recently. The contrast is especially vivid for cancer, where those diagnosed in the previous 0–1 years experienced an unintentional weight loss of 10% that was more than double the frequency among those diagnosed 2+ years earlier.

**Table 3:** Weight loss intentionality among individuals losing at least 10% of their body weight in the last year.^a^ Adults 30+, NHANES 1999–2014. 95% confidence interval in brackets.

Diagnosis	Yrs since diagnosis	% losing 10% or more…		% not losing 10%^a^	N
		Unintentionally^b^	Intentionally		
					
Entire sample	—	2.49 [2.26,2.72]	5.00 [4.65,5.35]	92.51 [92.08,92.95]	31,860
					
No diagnosis^c^	—	1.48 [1.26,1.71]	4.24 [3.81,4.67]	94.28 [93.78,94.78]	16,439
					
Arthritis	0–1	3.76 [2.55,4.97]	6.13 [4.45,7.80]	90.11 [88.15,92.08]	1,577
	2^+^	4.03 [3.48,4.59]	5.80 [5.12,6.48]	90.16 [89.27,91.06]	8,402
Cancer^d^	0–1	11.33 [6.61,16.05]	7.04 [3.10,10.98]	81.63 [75.21,88.04]	393
	2^+^	4.33 [3.13,5.53]	5.65 [4.26,7.04]	90.02 [88.12,91.91]	1,929
					
CVD^d^	0–1	9.00 [6.05,11.94]	6.06 [3.69,8.42]	84.95 [81.56,88.33]	693
	2^+^	5.56 [4.46,6.66]	5.79 [4.72,6.85]	88.65 [87.19,90.12]	3,065
					
Diabetes	0–1	5.94 [3.32,8.57]	12.3 [8.25,16.36]	81.75 [77.09,86.41]	525
	2^+^	4.89 [3.97,5.80]	9.40 [8.02,10.79]	85.71 [84.29,87.13]	3,688
					
Liver condition	0–1	10.69 [5.56,15.82]	8.25 [3.72,12.78]	81.06 [74.34,87.78]	241
	2^+^	5.09 [3.52,6.66]	5.49 [3.48,7.50]	89.42 [87.06,91.78]	1,044
					
Respiratory disease^d^	0–1	8.62 [3.00,14.23]	4.86 [1.85,7.86]	86.53 [80.59,92.46]	258
	2^+^	5.50 [4.33,6.67]	5.49 [4.38,6.61]	89.00 [87.44,90.57]	2,129

a. Only those losing at least 10 lbs. in the last year were asked about intentionality. Three respondents in the sample lost at least 10% of their body weight, but not 10 lbs., and were not asked about intention. They are included in the “did not lose 10%” category.

b. Weight loss for two respondents reporting “don’t know” for whether weight loss was intentional coded as unintentional weight loss.

c. None of the included illnesses diagnosed prior to study.

d. Cancer includes all cancers except non-melanoma skin cancers. CVD (cardiovascular disease) includes congestive heart failure, coronary heart disease, angina or angina pectoris, and stroke. Respiratory disease includes emphysema and chronic bronchitis.

Those diagnosed with any disease had a higher proportion intentionally losing at least 10% at both durations 0–1 and 2+ years than those without any diagnosis. The frequency of intentional weight loss was exceptionally high for those diagnosed with diabetes; the two highest values of intentional weight loss in the Table pertain to those diagnosed with diabetes at durations 0–1 and 2+.

S1 Table presents the same data as Tables 1 and 2 but describes the percent of the population in each weight change category who have a particular diagnosis. Among all those losing 10% or more, 56.7% had ever been diagnosed with one of these diseases, compared to 41.2% among those maintaining their weight within 5%. So if an individual has lost 10% or more of his or her weight in the past year, it is a reasonable supposition that he or she has been diagnosed with one of these diseases.

Tables 1–3 document weight change associated with illness diagnoses, regardless of the presence of other diagnoses. To isolate the weight change associated with each diagnosis net of multimorbidities, we estimate a multinomial logistic regression predicting the probability of experiencing a weight loss or gain of at least 10% in the past year, while controlling the presence of other diagnoses. These models are presented in Table 4. With the exception of respiratory diseases, those diagnosed with any of the diseases are significantly more likely to lose 10% of their weight than those without a diagnosis, regardless of duration since diagnosis. In all cases, the effect on weight loss of being diagnosed 2+ years earlier is smaller than that of being diagnosed more recently. The odds ratios in Model 1a, controlling only for diagnosis, age, sex, and weight, are relatively little affected by the adjustments for race/ethnicity, educational attainment, and smoking in Model 2a. Consistent with earlier tables, the likelihood of weight loss is greatest for those recently diagnosed with cancer.

**Table 4:** Odds ratios from multinomial logistic regressions predicting 10% weight change in the last year (reference category is no change of at least 10%). Adults 30+, NHANES 1999–2014. 95% confidence interval in brackets. N = 31,860.

	Lost at least 10%		Gained at least 10%	
	Model 1a	Model 2a	Model 1b	Model 2b
Male (0/1)	0.466***	0.450***	0.407***	0.389***
	[0.414,0.525]	[0.398,0.510]	[0.366,0.452]	[0.350,0.433]
Age	0.988***	0.992***	0.963***	0.963***
	[0.984,0.992]	[0.987,0.996]	[0.959,0.967]	[0.959,0.968]
Average wt (kg)	1.017***	1.018***	1.003*	1.002
	[1.014,1.020]	[1.015,1.021]	[1.000,1.005]	[1.000,1.005]
**Yrs since diagnosis** (ref: no diagnosis)				
** Arthritis**				
** **0–1	1.332*	1.285*	1.071	1.004
	[1.045,1.699]	[1.007,1.641]	[0.790,1.453]	[0.738,1.365]
** **2+	1.247***	1.187**	1.233**	1.148
	[1.110,1.400]	[1.057,1.333]	[1.062,1.432]	[0.987,1.336]
** Cancer**^a^				
** **0–1	3.061***	3.152***	1.052	1.061
	[1.935,4.841]	[1.990,4.991]	[0.573,1.931]	[0.576,1.953]
** **2+	1.306*	1.317*	1.111	1.142
	[1.048,1.627]	[1.061,1.634]	[0.831,1.486]	[0.855,1.525]
** CVD**^a^				
** **0–1	2.202***	2.140***	1.940**	1.790**
	[1.634,2.969]	[1.583,2.893]	[1.304,2.887]	[1.202,2.665]
** **2+	1.476***	1.405***	1.598***	1.456***
	[1.244,1.751]	[1.184,1.667]	[1.319,1.934]	[1.202,1.764]
** Diabetes**				
** **0–1	2.353***	2.252***	1.229	1.126
	[1.644,3.367]	[1.570,3.231]	[0.711,2.126]	[0.649,1.956]
** **2+	1.843***	1.786***	1.406**	1.284*
	[1.569,2.164]	[1.523,2.095]	[1.147,1.725]	[1.053,1.565]
** Liver condition**				
** **0–1	2.291***	2.212**	1.259	1.189
	[1.444,3.636]	[1.381,3.543]	[0.667,2.376]	[0.643,2.198]
** **2+	1.374*	1.338*	1.052	1.041
	[1.054,1.792]	[1.025,1.747]	[0.767,1.443]	[0.760,1.425]
** Resp. disease**^a^				
** **0–1	1.853*	1.592	3.304***	2.737**
	[1.051,3.267]	[0.925,2.741]	[1.820,5.998]	[1.503,4.985]
** **2+	1.241**	1.135	1.518***	1.383**
	[1.054,1.462]	[0.960,1.342]	[1.233,1.869]	[1.118,1.710]
**Smoke** (ref: never)^a^				
** **Former		1.015		1.367***
		[0.879,1.171]		[1.195,1.563]
** **Current		1.668***		1.502***
		[1.438,1.935]		[1.316,1.715]
**Race** (ref: NH white)				
** **NH black		1.054		1.456***
		[0.919,1.209]		[1.258,1.685]
** **Hispanic		1.128		1.218*
		[0.975,1.304]		[1.034,1.434]
** **Other		0.959		0.875
		[0.753,1.221]		[0.667,1.147]
**Educ** (ref: HS)				
** **<High school		1.122		1.132
		[0.965,1.305]		[0.967,1.324]
** **Some college		1.048		0.884
		[0.904,1.216]		[0.766,1.021]
** **BA+		0.778**		0.461***
		[0.653,0.928]		[0.390,0.546]

Stars denote statistical significance:

*** p < .001

** p < .01

* p < .05

a. Cancer includes all cancers except non-melanoma skin cancers. CVD (cardiovascular disease) includes congestive heart failure, coronary heart disease, angina or angina pectoris, and stroke. Respiratory disease includes emphysema and chronic bronchitis.

Table 4 also shows the parameters of regressions predicting the probability of having a weight gain of at least 10% in the past year. That probability was highest for those diagnosed with respiratory disease in the past 0–1 years. The probability of weight gain was also significant among those diagnosed with cardiovascular disease, and both diagnoses result in continued weight gain for those diagnosed 2+ years ago. Introducing smoking and sociodemographic variables in Model 2b substantially reduced the odds ratios for respiratory disease and cardiovascular disease, but the weight gains associated with these conditions remained significant. Being diagnosed with cardiovascular disease is unusually disruptive to weight maintenance since it is significantly associated with the likelihood of both gaining and losing weight. Additionally, blacks are significantly more likely to gain weight while people with bachelor’s degrees are much less likely to gain weight.

We added six interactive variables to each of the models in Table 4, representing combinations of diagnosis in the last year and mean weight. Of the 24 coefficients of variables expressing the interaction between weight and diagnosis, two were significant at 5%: among people recently diagnosed with liver disease, people of higher weight were less likely to gain weight, with and without controls for smoking, race/ethnicity, and educational attainment. Similarly, we added six interactive variables to the models in Table 4 representing combinations of a recent diagnosis and linear age. None of the 24 coefficients of variables representing these interactions for weight gain or weight loss was significant at 5%. These results are available from the authors upon request.

Although the models in Table 4 estimate weight change associated with specific diagnoses net of other conditions, they do not account for possible interactions between conditions. In a sensitivity analysis (not shown), we add variables to Models 1a and 1b indicating whether a respondent had ever been diagnosed with some of the most common comorbidities (arthritis and cardiovascular disease; cardiovascular disease and diabetes; arthritis and diabetes). Comorbidity odds ratios (ref: never diagnosed with both) for the first two comorbidities did not statistically differ from one, nor did their inclusion improve model fit. The odds ratio for having ever been diagnosed with arthritis and diabetes was significantly less than 1 at a level of p < .05 in both the weight loss and gain models, indicating that the odds of weight change associated with arthritis and diabetes alone are less than multiplicative for individuals diagnosed with both conditions.

S2 Table presents equivalent information to that in Table 4 but predicts an individual’s probability of gaining or losing at least 5% of their weight in the past year, rather than at least 10%. The same patterns emerge: all diagnoses except respiratory disease are associated with a significantly increased incidence of weight loss at 0–1 years. Odds ratios are also above 1 at 2+ years since diagnosis, though not consistently at a level of statistical significance. Diabetes, CVD, and respiratory disease were associated with significantly greater probabilities of weight gain of at least 5% at both durations 0–1 and 2+.

## Summary and discussion

Among changes in the health characteristics of the American population in the past several decades, rising levels of body mass index and obesity stand out as especially problematic.[13] While changes in the prevalence of various BMI categories have been effectively documented,[14,15] details of the annual changes in weight that produce changes in the weight distribution have been neglected. In this study, we investigate the role of disease incidence in annual changes in weight.

Results in Table 2 show that disease is playing a major role in population weight change. Adults who have not been diagnosed with one of the six diseases that we study gain weight at the very fast pace of 0.55%/year. However, the gain for the population as a whole is only 0.16%/year because of net weight loss among those diagnosed with a disease. The probability of losing at least 10% of weight in the past year ranges from 9.9% to 18.9% for those diagnosed in the past 0–1 years with one of the six diseases, compared to only 5.7% for those with no diagnosis. The same pattern of excess weight loss associated with disease is evident for the population diagnosed 2+ years ago (Table 2). Furthermore, relative to those with no diagnosis, those with any of the six disease diagnoses were much more likely to *unintentionally* lose 10% of their body weight in the past year (Table 3). Our results reveal that disease incidence is playing a major role in US patterns of weight change, a role that has not previously been identified.

Our study distinguished between voluntary and involuntary weight change as these two mechanisms have different clinical implications. Involuntary weight loss is an important prognostic indicator of disease onset and progression and evidence on patterns of involuntary weight change associated with disease diagnoses may be useful to health care providers involved in managing patient care. Evidence on patterns of voluntary weight loss associated with a disease diagnosis, in contrast, provide insight into the extent to which patients undergo behavioral modification following diagnosis of a new condition. Our finding that rates of voluntary weight loss were greater following a diagnosis of diabetes compared to a new diagnosis of other conditions suggests that there are opportunities for more aggressive weight-loss counseling in the context of other diseases. Whereas patients may recognize the benefits of weight loss for management of diabetes, they may be less aware of the benefits of weight loss for improving prognosis in other diseases such as CVD and cancer.

These results have important implications for studies of the relation between BMI and mortality. The persistent weight loss among people diagnosed with various diseases is likely related to the frequent observation in cohort studies that those who lose weight are subject to higher mortality than those who retain their weight.[16–20] It may also help to explain why some prior studies have found a mortality advantage in overweight and/or obesity categories relative to normal weight status.[21–23] This finding is often considered paradoxical because mortality is positively associated with baseline body mass indexes above the normal range.[24,25] The fact that so many of those losing weight are ill helps to explain the paradox.[26–28] We estimate that 56.7% of adults aged 30+ who have lost at least 10% of their weight in the past year have been diagnosed with one of the six disease categories that we consider. The extent of illness-associated weight loss is clearly capable of leaving a substantial imprint on relations between weight and mortality. That imprint should be especially clear among those who are losing weight unintentionally.[29–31] Unless this possibility of reverse causation is satisfactorily addressed in a study’s research design, disease-associated weight loss is likely to be an important source of downward bias in estimates of the excess mortality associated with obesity.

This study has several strengths and weaknesses. It is the first comparative study of weight changes associated with the diagnosis of various illnesses. It employs a nationally representative sample that achieves a substantial sample size by aggregating multiple waves. A weakness is that weight changes are self-reported. While self-reports of body weight are highly correlated with actual weight, systematic patterns of underreporting have been identified.[32] Whether these biases “net out” when reporting weight change over the past year is unknown. The diagnoses of diseases are also self-reported and not a direct product of clinical measurements. The category with no diagnoses likely includes individuals with undiagnosed conditions, as well as individuals living with other conditions not considered in our analysis. The presence of ill individuals in the “none” category would likely bias downwards our estimates of relative weight change associated with particular conditions. An additional weakness is that weight changes are only reported for people who have survived to the survey. Alley et al.[33] describe an accelerating pattern of weight loss before death, from which we may infer that survivors with a particular diagnosis are less likely to have lost weight than members of their cohort who have died with that diagnosis. Additionally, NHANES surveys only sample the non-institutionalized population. The institutionalized population may have patterns of weight change associated with illness that differ from those of the non-institutionalized. A final weakness is that the relationships observed cannot be unambiguously interpreted as causal. For those diagnosed in the last year, weight change in the last year may have precipitated a doctor’s visit, and thus a diagnosis. The exact timing of weight change and disease diagnosis is not critical for the associations established in this paper.

## Conclusion

Disease incidence is playing a major role in patterns of weight change in the American population. On average, those diagnosed with arthritis, cancer, cardiovascular disease, diabetes, or a liver condition lose weight 0–1 years after diagnosis and they continue to lose weight 2+ years after diagnosis. These weight losses are substantial enough that they offset most of the annual weight gain that would be observed in the US population in the absence of these diseases.

Those diagnosed with these conditions, as well as respiratory disease, are more likely to experience unintentional weight losses of at least 10% than individuals with no diagnoses. Those diagnosed with cancer have an exceptionally high frequency of unintentional weight loss. The patterns presented in this paper, especially those associated with unintentional weight loss, may serve as useful standards for clinicians considering how weight changes observed in individual patients may compare to those in the population as a whole.

## Supporting information

S1 Table Percent of population in each weight change category who have a particular diagnosis, by years since diagnosis.^a^ Adults 30+, NHANES 1999–2014. Standard errors in parentheses.
(DOCX)

S2 Table Odds ratios from multinomial logistic regressions predicting 5% weight change in the last year (reference category is no change of at least 5%). Adults 30+, NHANES 1999–2014. 95% confidence interval in brackets. N = 31,860.
(DOCX)
