# Supplementary material for: Patterns of weight change associated with disease diagnosis in a national sample
Source: PLoS One. 2018 Nov 26;13(11):e0207795. doi: 10.1371/journal.pone.0207795 (PMC6261267; doi:10.1371/journal.pone.0207795)
Supplement: S1 Table — Standard errors in parentheses. (DOCX) [file pone.0207795.s001.docx]

**S1 Table.** Percent of population in each weight change category who have a particular diagnosis, by years since diagnosis.^a^ Adults 30+, NHANES 1999-2014. Standard errors in parentheses.

|  | Weight loss in last year | | Maintained weight within 5% in last year | Weight gain in last year | |
| --- | --- | --- | --- | --- | --- |
|  | 10% or more | 5% or more |  | 5% or more | 10% or more |
|  |  |  |  |  |  |
| No diagnosis^b^ | 43.29 (1.28) | 47.61 (0.91) | 58.77 (0.58) | 57.56 (0.86) | 55.32 (1.27) |
|  |  |  |  |  |  |
|  |  |  |  |  |  |
| Diagnosed 0-1 years ago | |  |  |  |  |
|  |  |  |  |  |  |
| Arthritis | 5.81 (0.62) | 5.42 (0.39) | 4.28 (0.17) | 3.83 (0.31) | 4.04 (0.57) |
|  |  |  |  |  |  |
| Cancer^c^ | 2.57 (0.49) | 1.89 (0.26) | 0.84 (0.07) | 0.97 (0.17) | 0.79 (0.25) |
|  |  |  |  |  |  |
| CVD^c^ | 3.26 (0.41) | 2.55 (0.24) | 1.34 (0.10) | 1.74 (0.20) | 1.89 (0.33) |
|  |  |  |  |  |  |
| Diabetes | 3.04 (0.43) | 2.67 (0.25) | 0.87 (0.08) | 1.20 (0.18) | 1.17 (0.28) |
|  |  |  |  |  |  |
| Liver conditions | 1.55 (0.29) | 1.09 (0.16) | 0.46 (0.06) | 0.68 (0.13) | 0.70 (0.20) |
|  |  |  |  |  |  |
| Respiratory disease | 1.34 (0.31) | 0.90 (0.14) | 0.60 (0.08) | 1.13 (0.23) | 1.81 (0.47) |
|  |  |  |  |  |  |
|  |  |  |  |  |  |
| Diagnosed 2+ years ago | |  |  |  |  |
|  |  |  |  |  |  |
| Arthritis | 31.57 (1.18) | 29.3 (0.77) | 22.61 (0.47) | 24.06 (0.72) | 24.11 (1.11) |
|  |  |  |  |  |  |
| Cancer^c^ | 7.69 (0.72) | 6.36 (0.43) | 5.73 (0.21) | 5.31 (0.46) | 5.49 (0.74) |
|  |  |  |  |  |  |
| CVD^c^ | 11.04 (0.70) | 9.14 (0.43) | 7.00 (0.26) | 6.47 (0.38) | 7.45 (0.62) |
|  |  |  |  |  |  |
| Diabetes | 15.77 (0.82) | 13.08 (0.53) | 7.23 (0.23) | 7.21 (0.33) | 8.59 (0.67) |
|  |  |  |  |  |  |
| Liver conditions | 4.45 (0.50) | 3.75 (0.31) | 2.93 (0.16) | 3.40 (0.30) | 3.10 (0.44) |
|  |  |  |  |  |  |
| Respiratory disease | 9.99 (0.69) | 8.60 (0.49) | 5.79 (0.25) | 8.83 (0.48) | 9.85 (0.79) |
|  |  |  |  |  |  |
| N | 2,515 | 5,918 | 20,449 | 5,493 | 2,444 |

a. Columns do not add to 100% since some individuals have been diagnosed with multiple conditions.

b. None of the included conditions diagnosed prior to study.

c. Cancer includes all cancers except non-melanoma skin cancers. CVD includes congestive heart failure, coronary heart disease, angina or angina pectoris, and stroke. Respiratory disease includes emphysema and chronic bronchitis.
