# Supplementary material for: Patterns of weight change associated with disease diagnosis in a national sample
Source: PLoS One. 2018 Nov 26;13(11):e0207795. doi: 10.1371/journal.pone.0207795 (PMC6261267; doi:10.1371/journal.pone.0207795)
Supplement: S2 Table — Adults 30+, NHANES 1999–2014. 95% confidence interval in brackets. N = 31,860. (DOCX) [file pone.0207795.s002.docx]

**S2 Table.** Odds ratios from multinomial logistic regressions predicting 5% weight change in the last year (reference category is no change of at least 5%). Adults 30+, NHANES 1999-2014. 95% confidence interval in brackets. N=31,860.

|  | **Lost at least 5%** | |  | **Gained at least 5%** | |  |
| --- | --- | --- | --- | --- | --- | --- |
|  | Model 1a | Model 2a |  | Model 1b | Model 2b |  |
| Male (0/1) | 0.488^***^ | 0.477^***^ |  | 0.468^***^ | 0.458^***^ |  |
|  | [0.454,0.525] | [0.441,0.515] |  | [0.434,0.504] | [0.425,0.493] |  |
|  |  |  |  |  |  |  |
| Age | 0.991^***^ | 0.994^***^ |  | 0.969^***^ | 0.970^***^ |  |
|  | [0.988,0.994] | [0.990,0.997] |  | [0.966,0.972] | [0.967,0.973] |  |
|  |  |  |  |  |  |  |
| Average wt (kg) | 1.017^***^ | 1.017^***^ |  | 1.002^*^ | 1.002 |  |
|  | [1.015,1.019] | [1.015,1.019] |  | [1.000,1.005] | [0.999,1.004] |  |
|  |  |  |  |  |  |  |
| **Yrs since diagnosis** (ref: no diagnosis) | |  |  |  |  |  |
| **Arthritis** |  |  |  |  |  |  |
| 0-1 | 1.269^*^ | 1.238^*^ |  | 1.040 | 0.999 |  |
|  | [1.055,1.526] | [1.027,1.494] |  | [0.869,1.245] | [0.832,1.200] |  |
| 2+ | 1.232^***^ | 1.198^***^ |  | 1.286^***^ | 1.238^***^ |  |
|  | [1.126,1.347] | [1.094,1.312] |  | [1.157,1.430] | [1.114,1.376] |  |
| **Cancer**^a^ |  |  |  |  |  |  |
| 0-1 | 2.481^***^ | 2.539^***^ |  | 1.465 | 1.491 |  |
|  | [1.794,3.432] | [1.837,3.508] |  | [0.979,2.193] | [0.990,2.245] |  |
| 2+ | 1.052 | 1.062 |  | 1.047 | 1.075 |  |
|  | [0.886,1.250] | [0.895,1.261] |  | [0.865,1.268] | [0.887,1.303] |  |
| **CVD**^a^ |  |  |  |  |  |  |
| 0-1 | 1.883^***^ | 1.854^***^ |  | 1.838^***^ | 1.761^***^ |  |
|  | [1.457,2.435] | [1.428,2.408] |  | [1.396,2.421] | [1.328,2.334] |  |
| 2+ | 1.218^**^ | 1.176^*^ |  | 1.282^**^ | 1.212^*^ |  |
|  | [1.070,1.387] | [1.034,1.338] |  | [1.102,1.491] | [1.041,1.411] |  |
| **Diabetes** |  |  |  |  |  |  |
| 0-1 | 2.686^***^ | 2.567^***^ |  | 1.569^*^ | 1.463^*^ |  |
|  | [2.031,3.554] | [1.930,3.413] |  | [1.082,2.275] | [1.003,2.134] |  |
| 2+ | 1.636^***^ | 1.574^***^ |  | 1.196^**^ | 1.106 |  |
|  | [1.447,1.850] | [1.389,1.783] |  | [1.050,1.363] | [0.968,1.262] |  |
| **Liver condition** |  |  |  |  |  |  |
| 0-1 | 1.916^***^ | 1.843^**^ |  | 1.433 | 1.359 |  |
|  | [1.325,2.771] | [1.271,2.673] |  | [0.929,2.210] | [0.883,2.091] |  |
| 2+ | 1.232 | 1.204 |  | 1.222 | 1.209 |  |
|  | [0.999,1.519] | [0.975,1.488] |  | [0.987,1.512] | [0.976,1.498] |  |
| **Resp. disease**^a^ |  |  |  |  |  |  |
| 0-1 | 1.261 | 1.125 |  | 1.918^**^ | 1.685^*^ |  |
|  | [0.820,1.939] | [0.744,1.703] |  | [1.186,3.103] | [1.035,2.745] |  |
| 2+ | 1.231^**^ | 1.155 |  | 1.478^***^ | 1.397^***^ |  |
|  | [1.062,1.425] | [0.999,1.336] |  | [1.260,1.733] | [1.194,1.635] |  |
| **Smoke** (ref: never) | |  |  |  |  |  |
| Former |  | 1.058 |  |  | 1.210^***^ |  |
|  |  | [0.951,1.177] |  |  | [1.107,1.323] |  |
| Current |  | 1.520^***^ |  |  | 1.320^***^ |  |
|  |  | [1.359,1.701] |  |  | [1.191,1.465] |  |
| **Race** (ref: NH white) | |  |  |  |  |  |
| NH black |  | 1.255^***^ |  |  | 1.461^***^ |  |
|  |  | [1.139,1.383] |  |  | [1.319,1.617] |  |
| Hispanic |  | 1.234^***^ |  |  | 1.311^***^ |  |
|  |  | [1.115,1.366] |  |  | [1.156,1.487] |  |
| Other |  | 0.936 |  |  | 0.885 |  |
|  |  | [0.781,1.121] |  |  | [0.731,1.070] |  |
| **Educ** (ref: HS) |  |  |  |  |  |  |
| <High school |  | 1.038 |  |  | 0.996 |  |
|  |  | [0.924,1.166] |  |  | [0.875,1.133] |  |
| Some college |  | 1.079 |  |  | 0.941 |  |
|  |  | [0.973,1.196] |  |  | [0.845,1.048] |  |
| BA+ |  | 0.843^**^ |  |  | 0.582^***^ |  |
|  |  | [0.747,0.951] |  |  | [0.509,0.664] |  |

Stars denote statistical significance: *** p<.001; ** p<.01; * p<.05

a. Cancer includes all cancers except non-melanoma skin cancers. CVD (cardiovascular disease) includes congestive heart failure, coronary heart disease, angina or angina pectoris, and stroke. Respiratory disease includes emphysema and chronic bronchitis.
